# Microblog discourse analysis for parenting style assessment

**DOI:** 10.3389/fpubh.2025.1505825

**Published:** 2025-02-11

**Authors:** Zihan Wei, Lei Cao, Zhihong Qiao, Fang Luo, Xin Wang, Junrui Tian, Qi Li

**Affiliations:** ^1^School of Economics and Management, Beijing University of Chemical Technology, Beijing, China; ^2^Faculty of Psychology, Beijing Normal University, Beijing, China; ^3^Department of Engineering Science, University of Oxford, Oxford, United Kingdom; ^4^Department of Computer Science and Technology, Tsinghua University, Beijing, China

**Keywords:** parenting style, microblog discourse, deep learning, parenting style dataset, social media

## Abstract

**Introduction:**

Parents' negative parenting style is an important cause of anxiety, depression, and suicide among university students. Given the widespread use of social media, microblogs offer a new and promising way for non-invasive, large-scale assessment of parenting styles of students' parents.

**Methods:**

In this study, we have two main objectives: (1) investigating the correlation between students' microblog discourses and parents' parenting styles and (2) devising a method to predict students' parenting styles from their microblog discourses. We analyzed 111,258 posts from 575 university students using frequency analysis to examine differences in the usage of topical and emotional word across different parenting styles. Informed by these insights, we developed an effective parenting style assessment method, including a correlation injection module.

**Results:**

Experimental results on the 575 students show that our method outperforms all the baseline NLP methods (including ChatGPT-4), achieving good assessment performance by reducing MSE by 14% to 0.12.

**Discussion:**

Our study provides a pioneering microblog-based parenting style assessment tool and constructs a dataset, merging insights from psychology and computational science. On the one hand, our study advances the understanding of how parenting styles are reflected in the linguistic and emotional expressions of students on microblogs. On the other hand, our study provides an assisting tool that could be used by healthcare institutions to identify students' parenting styles. It facilitates the identification of suicide risk factors among microblog student users, and enables timely interventions to prevent suicides, which enhances human wellbeing and saves lives.

## 1 Introduction

Parenting style is the summary of the characteristics of the parents' various parenting behaviors, attitudes, and emotions. The concept of parenting style has been mainly divided into three orientations: dimensional orientation, practical behavior orientation, and comprehensive orientation (1). First, the dimensional orientation considers that the parenting style is a relatively fixed parenting behavior tendency shown by parents in daily life, including the speech and emotion between parents and children (2). The most representative example of this orientation is the classification of parenting as authoritative, permissive, or authoritarian (3). Authoritative parents not only have high demands and expectations for their children but also provide warm emotional support and responses (3). They encourage their children to express their thoughts and focus on cultivating their children's autonomy (3). Permissive parents grant their children a great deal of freedom, set few rules and requirements, and are overly tolerant of their children (3). Authoritarian parents, on the other hand, emphasize absolute obedience, establish strict rules, yet lack emotional warmth and communication (3). Snow et al. (4) further enriched the theory by adding the neglectful parenting style. Neglectful parents lack both affection for their children and the establishment of rules and requirements. This parenting style often has severe negative impacts on children's growth (4). Second, the practical behavior orientation considers the parenting style as the specific behavior of parents when raising their children, such as the time spent accompanying their children and the extent of their involvement in their children's educational activities (5). Finally, the comprehensive orientation integrates the first two definitions. This means that parenting style is not only a summary of the characteristics of the parents' various parenting behaviors but also includes parenting attitudes and emotions.

A negative parenting style is characterized by low emotional warmth and high rejection and overprotection (6), which has been identified as an important cause of anxiety, depression, and suicide among university students (7, 8). Assessment of the parenting style of people's parents allows healthcare institutions to pay attention to people with mild anxiety, mild depression, and potential suicidal ideation in a timely manner and make appropriate interventions to prevent the situation from worsening.

Currently, there is a lack of automated assessment methods for parenting styles. Traditional assessment methods based on questionnaires and interviews often require individuals to complete a professional questionnaire or participate in a face-to-face interview with a psychology expert. In 1980, Perris and Jacobsson (9) research, proposed the parental rearing behavior questionnaire (EMBU). The questionnaire has 15 subscales with 81 items. The 15 subscales form three father-related factors and four mother-related factors. To make this scale suitable for the Chinese population, Yue et al. (10) proposed the Chinese version of EMBU by revising the EMBU. The revised questionnaire contains a sub-questionnaire for both the father and the mother. The father's sub-questionnaire comprises 58 items in six dimensions: *Emotional warmth, Excessive Interference, Punishment, Rejection, Overprotection*, and *Preference*. The mother's sub-questionnaire comprises 57 items in five dimensions: *Emotional warmth, Excessive Interference, Punishment, Rejection*, and *Preference*. Although this questionnaire provide a comprehensive assessment of parenting styles, it may be time-consuming for respondents. Later, Jiang et al. (11) further introduced the short-form Chinese version of EMBU (s-EMBU-C) by reducing the number of items from 115 to 42. The short-form questionnaire only has three dimensions: *Rejection, Emotional warmth*, and *Overprotection*. This questionnaire was tested among the Chinese university students, and the results show that it has good reliability and validity.

However, this type of invasive method requires high enthusiasm from individuals and is difficult to apply on a large scale. Medical and healthcare institutions lack low-cost, automated, and non-invasive methods. With the development of social media, increasingly more people are expressing their inner feelings on social media (e.g., on microblogs) (12, 13). Having the advantages of being large-scale, low-cost, and open, microblogs allow us to access the parenting styles of a large number of students' parents in a no-contact manner.

However, limited research exists in the literature addresses the assessment of parenting styles of students' parents based on microblogs. The first challenge is that the correlation between students' discourses on microblogs and parents' parenting styles has been less explored. Moreover, in the long-time series analysis of student users, there is often a lot of noisy data, and critical information can be overwhelmed by numerous meaningless posts.

**Figure 1:**
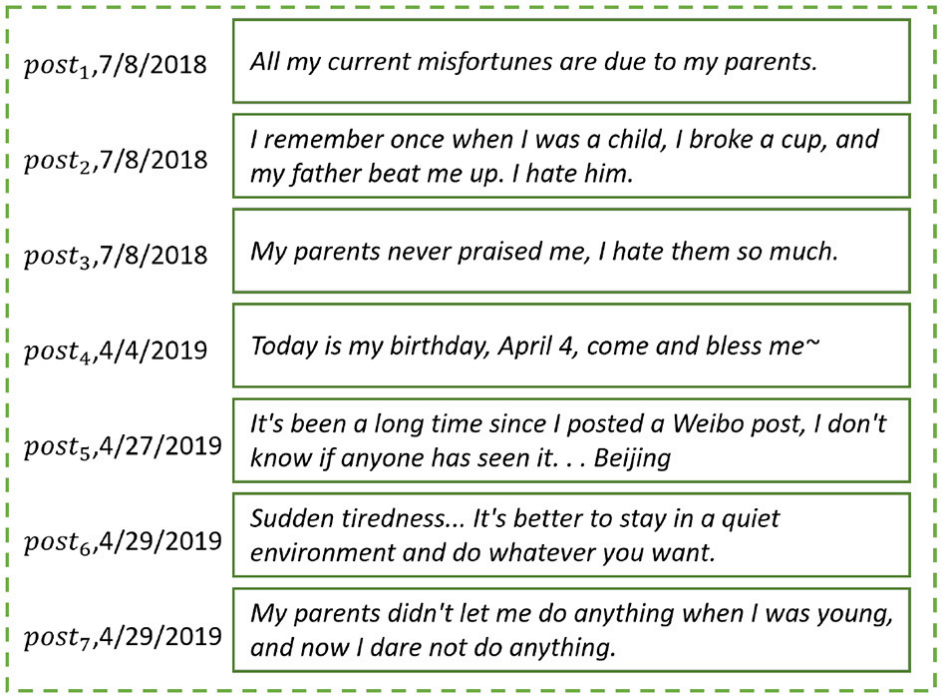
Real open post sequence of a university student user from May 1, 2018 to April 30, 2019.

Figure 1 shows a real open post sequence written by a student on a microblog over the period of a year. We make the following observations.

(*Observation 1*) People may talk about their childhood experiences of being parented on microblogs. According to the described experiences, the student's parents may have beaten her for small things, seldom praised her, and seldom let her do what she wanted when she was young. These descriptions indicate that the student grew up under a negative parenting style.

(*Observation 2*) Noisy information that is not related to the parenting style appears in the student's open post sequence, such as in the student's comments on daily trivia.

The above observations inspire us to assess the parenting styles of student microblog users' parents based on their discourses.

In this study, we propose to (1) first investigate the correlation between students' discourses on microblogs and their parents' parenting styles and (2) then predict the parenting style of the microblog student users' parents based on the found correlation and their discourses.

**Subtask 1: exploration of the correlation between microblog student user's discourse and parents' parenting style**

Subtask 1 is not only designed to explore the correlation between the discourse from student's open posts and the parenting style of their parents, but it also plays a crucial role in understanding the differences in emotional and linguistic expressions across various parenting styles.

To address the subtask 1, we first constructed a microblog-based parenting style dataset, which contains numerous discourses from microblog student users. The dataset contains 111,258 open posts made by 575 students from November 1, 2019, to October 31, 2022. Subsequently, the associations between the discourses and three types of parenting styles were investigated from the perspectives of students' linguistic and emotional expressions.

Through data analysis, we found that positive, mixed, and negative parenting styles show significant differences in linguistic expressions and emotional expressions on microblogs. In this study, linguistic and emotional expressions were analyzed by examining the frequency of different topics and emotional words used by students. This approach allowed us to identify distinct patterns of expression associated with each parenting style. Under a negative parenting style, the effect of gender on negative emotional expressions is more significant than that under a positive or mixed parenting style.

**Subtask 2: microblog-based parenting style assessment**

Subtask 2 focuses on inferring a student's parents' parenting style, primarily by utilizing the correlation between the student's discourse and their parents' parenting style. This correlation, discovered through our analysis, plays a crucial role in providing a more accurate assessment of the parenting style, beyond just relying on microblog data alone.

The parenting style is represented in the form of scores in the dimensions *Rejection, Emotional warmth*, and *Overprotection* (11); i.e., {*s*_*r*_, *s*_*e*_, *s*_*o*_}. Let *P* = {*post*_1_, *post*_2_, ⋯ , *post*_*n*_} be a student's open post sequence. That is, *F*(*P*) = {*s*_*r*_, *s*_*e*_, *s*_*o*_}.

To solve subtask 2, we first used Sentence BERT (14) to extract the linguistic and emotional information from the student's open posts. An attention layer was then used to handle data noise and capture the key discourses describing the student's experiences in childhood. Subsequently, we designed a novel correlation injection layer to merge the found correlations and the above key discourses. Last, based on the correlations and key discourses, two fully-connected layers predicted the parenting style of the parents of the student as scores {*s*_*r*_, *s*_*e*_, *s*_*o*_}.

The parenting style dataset was used to evaluate the effectiveness of the method. Experimental results obtained on the dataset show that the parenting style assessment method effectively predicts the parenting style of the parents of the student with a mean square error (MSE) of 0.12 and mean absolute error (MAE) of 0.28, which outperforms all the baseline methods (e.g., ChatGPT-4).

In summary, this study makes the following contributions:

- Exploration of student's discourse and parenting style: We explore the correlation between microblog university student user's discourse and parents' parenting style from the perspectives of linguistic and emotional expressions. Compared with the students under the positive and mixed parenting styles, students under the negative parenting style tend to use more words related to “health,” “death,” and “love,” and express more negative emotions (i.e., “sad,” “fear,” and “hate”) in their discourses.
- Introduction of a microblog-based parenting style assessment method: We propose the first method of this kind. Performance study shows that this method can infer the parenting styles of students' parents with minor errors. This method facilitates the assessment of more suicide risk factors.
- Creation of a large microblog-based parenting style dataset: The dataset contains 111,258 open posts made by 575 students from November 1, 2019, to October 31, 2022. It facilitates subsequent research in the fields of psychology and computer science.

## 2 Materials and methods

In this section, we first explored the correlation between students discourses and parenting style types, and then designed a deep learning-based parenting style assessment method using students' discourses and found correlations.

### 2.1 An exploration of the correlation between discourse and parenting style type

#### 2.1.1 Construction of parenting style dataset

Due to the absence of a dataset related to parenting styles at present, to explore the relationship between students' discourses and the parenting styles they experienced, we constructed a new parenting style dataset. We constructed this dataset via Weibo, the largest social media platform in China. To recruit participants, we posted announcements in the “University Student Questionnaire Completion” topic on Weibo. This topic, which gathers over 127,000 university students across the country, is the largest community for questionnaire completion on the platform.

Participants were asked to fill out a questionnaire that included their parenting style, Weibo ID (for collecting their public microblog data later), age, gender, payment account details, and real name. The payment account and real name were collected solely for the purpose of disbursing participant fees. Each participant who completed the questionnaire carefully received a compensation of 5 yuan. We used Python code to collect their open microblog data to construct a parenting style dataset.

To ensure data reliability and prevent individuals from completing the questionnaire multiple times using different accounts, we restricted submissions to one per IP address.

For the parenting style assessment, we used the short-form Chinese version of the EMBU (s-EMBU-C) (11). This 21-item questionnaire is widely applied in China and is well-suited for Chinese participants. Specifically, this questionnaire exhibits high reliability and validity, demonstrating a high degree of dependability. Its developer administered the questionnaire to 700 Chinese college students. The test - retest reliability of this questionnaire, measured after 10 weeks, ranges from 0.70 to 0.81. In terms of validity, this questionnaire has a significant correlation with the corresponding dimensions of the Chinese version of EMBU, with the correlation coefficients all above 0.8. The questionnaire evaluates three parenting dimensions: *Rejection* (six items), *Emotional Warmth* (seven items), and *Overprotection* (eight items). Responses were rated on a four-point Likert scale, ranging from “never occurs” to “always occurs.” Scores for each dimension were calculated as the average of the respective items.

To protect participant privacy, we implemented several measures. First, participants' payment account details and real names were permanently deleted after fee disbursement. Second, public Weibo data was collected using participants' Weibo IDs, ensuring that no personal information was exposed. Additionally, participants were fully informed before participation that their public Weibo data would be used for scientific research purposes.

We received a total of 3,183 questionnaires over the period of a month. Approximately one-third of the users did not supply their microblog usernames and were removed because we could not confirm their authenticity. Among the remaining users, a user was regarded as a valid student user if he/she satisfied the following conditions: (1) He/she answered the polygraph question correctly. We added a simple polygraph question: “*For this question, please choose ‘occasionally occurs’ for your father and ‘always occurs’ for your mother*.” (2) He/she had between 5 and 5,000 followers. Too few followers meant that he/she was not active enough on microblog and too many followers meant that he/she may be an institution or a public figure. (3) He/she had made more than 10 original posts in the past year. Too few original posts indicated low activity on the microblog. The data collection process received approval from the local ethics committee, reference number: 202302220019. After filtering, there were 575 valid students.

#### 2.1.2 Description of the parenting style dataset

This dataset contains 575 microblog student users, including 281 males and 294 females. Each student was assigned three scores (*s*_*r*_, *s*_*e*_, and *s*_*o*_) for the dimensions of *Rejection, Emotional Warmth*, and *Overprotection*. The age distribution ranged from 14 to 51 years, with an average age of 24.0 years and a standard deviation of 5.8 years. We wrote a Python program to collect all the open posts of these students dated from November 1, 2019, to October 31, 2022. Finally, we got 111,258 open posts from 575 students. Each student on average has 193.5 posts. The maximum and minimum numbers of posts made by a student were 1987 and 10, respectively. To develop the parenting style assessment method, we divided the 575 students into a training set of 475 students, a validation set of 50 students, and a test set of 50 students.

**Figure 2:**
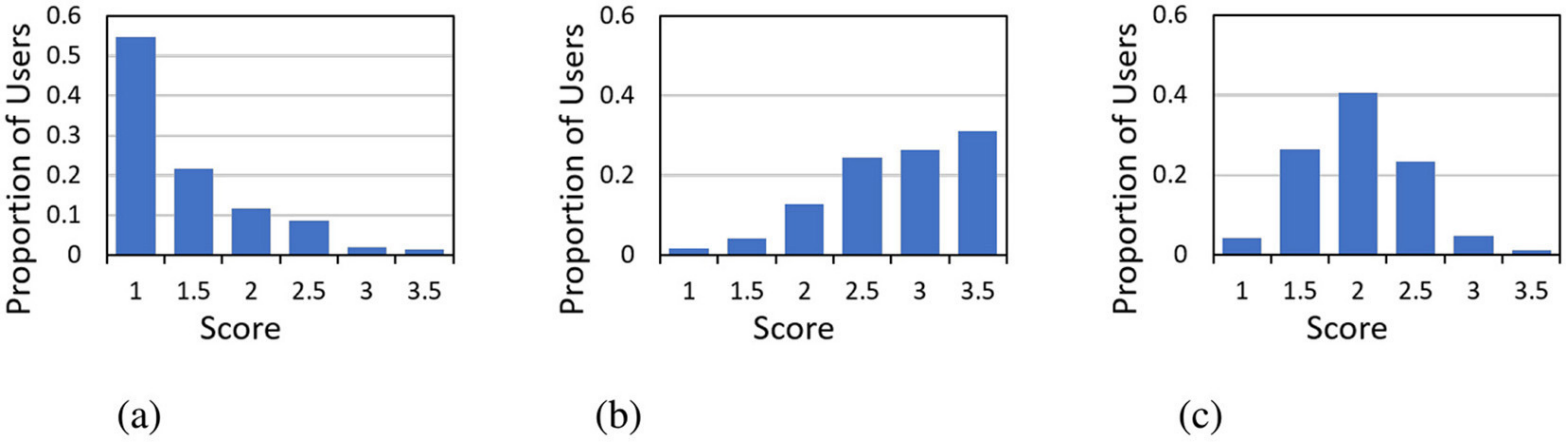
The distribution of the parenting scores of 575 microblog student users in the dimensions **(A)** Rejection, **(B)** Emotion warmth, and **(C)** Overprotection.

Figure 2 shows the distribution of parenting scores in the three dimensions. The average scores of the 575 students in the dimensions *Rejection, Emotion warmth*, and *Overprotection* were 1.59, 3.04, and 2.21, respectively.

Peng et al. (6) argued that parenting styles can be categorized into three types—positive, mixed, and negative—using Latent Profile Analysis (LPA). Following this approach, we classified the 575 students into these three types based on their scores in the dimensions of *Rejection* (*s*_*r*_), *Emotional warmth* (*s*_*e*_), and *Overprotection* (*s*_*o*_):

- Positive-parenting-style-type students. Students have higher scores in the dimension *Emotional warmth* and lower scores in the *Rejection* and *Overprotection*, i.e., (*s*_*e*_ > *s*_*r*_ + 1)&(*s*_*e*_ > *s*_*o*_ + 1).
- Mixed-parenting-style-type students. Scores in the dimension *Emotional warmth* are not significantly different from those in the dimensions *Rejection* and *Overprotection*, i.e., (|*s*_*e*_ − *s*_*r*_| < 1)&(|*s*_*e*_ − *s*_*o*_| < 1).
- Negative-parenting-style-type students. Students have lower scores in the dimension *Emotional warmth* and higher scores in the dimensions *Rejection* and *Overprotection*, i.e., (*s*_*e*_ + 1 < *s*_*r*_)&(*s*_*e*_ + 1 < *s*_*o*_).

**Figure 3:**
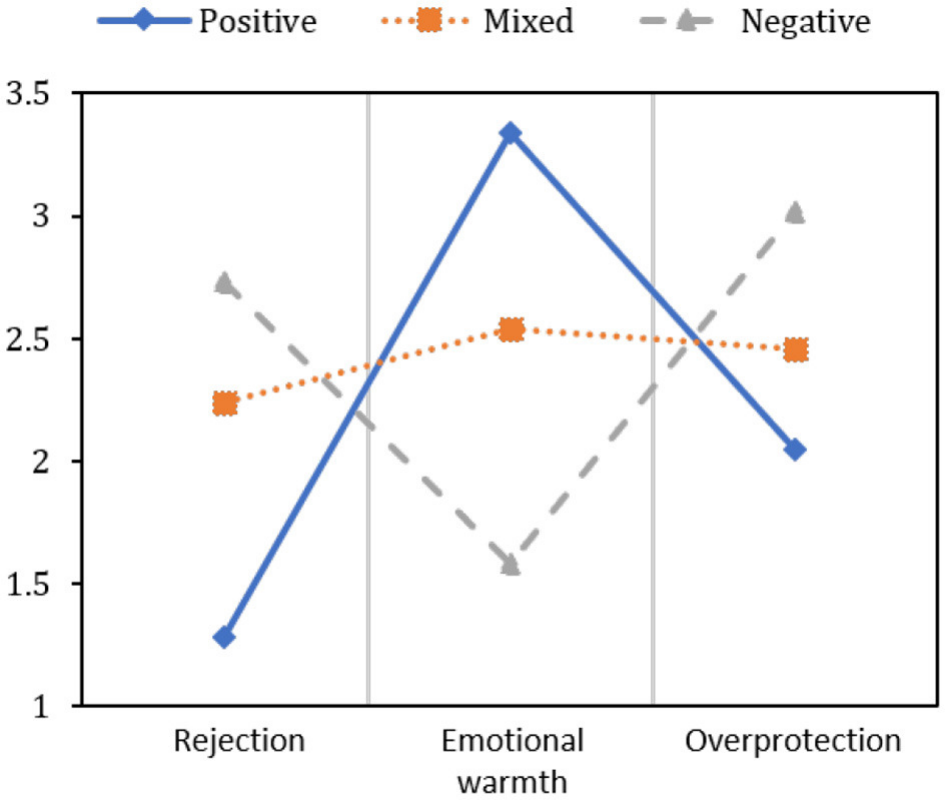
The average scores of the three parenting style types in the three dimensions.

There are 379 (66.4%), 162 (28.4%), and 16 (2.8%) students who grew up under positive, mixed, and negative parenting style types. Moreover, there are 14 (2.5%) students whose parenting style scores did not satisfy the above three types, e.g., (*s*_*r*_ + 1 < *s*_*e*_)&(*s*_*e*_ < *s*_*o*_). Those students were not considered in the following analysis. The statistical results are broadly in line with the statistical finding reported by Peng et al. (6) that the proportions of positive, mixed, and negative parenting style types are 69.1%, 22.3%, and 8.6%, respectively. Figure 3 shows the average scores of the three parenting style types in the dimensions *Rejection, Emotional warmth*, and *Overprotection*, respectively.

#### 2.1.3 Correlation exploration

We analyzed the difference between the three parenting style types from the perspectives of linguistic expressions and emotional expressions in discourses.

Table 1 lists the topic-related word frequencies used by the three types of students on microblogs. The word frequencies were calculated using the Simplified Chinese Linguistic Inquiry Word Count, TextMind (15). The corpus contains 72,748 open posts from positive-parenting-style-type students, 34,326 from mixed-parenting-style-type students, and 2,366 from negative-parenting-style-type students. There are 11 topics, i.e., “social,” “family,” “friend,” “health,” “work,” “leisure,” “money,” “religion,” “death,” “psychology,” and “love.” The differences between the maximum and secondary maximum word frequencies are significant (*p* < 0.01) for all topics. We found that compared with the other types of students, positive-parenting-style-type students were more prone to use words related to topics like “social,” “family,” “friend,” “work,” “leisure,” “money,” and “psychology.” Mixed-parenting-style-type students talked more about “religion.” Negative-parenting-style-type students used more words related to “health,” “death,” and “love.” Besides, “Social,” “work,” and “leisure” are the most frequently discussed topics.

**Table 1:** The mean proportions of topic-related words used by 575 microblog student users under the parenting style types “Positive,” “Mixed,” and “Negative.”

**Parenting style type**	**Social**	**Family**	**Friend**	**Health**	**Work**	**Leisure**	**Money**	**Religion**	**Death**	**Psychology**	**Love**
Positive	**5.64**	**0.56**	**0.26**	0.73	**4.20**	**4.56**	**1.78**	0.30	0.17	**1.73**	0.09
Mixed	5.09	0.39	0.14	0.64	4.12	4.01	1.53	**0.32**	0.24	1.65	0.10
Negative	5.12	0.49	0.13	**0.87**	3.80	2.37	0.90	0.27	**0.38**	1.70	**0.14**

All values are percentages (%). In each topic, the maximum proportion among the three types of students is shown in bold.

From the perspective of emotional expression (Table 2), we observe that students under the positive and mixed parenting style types wrote more posts with positive emotions (i.e., “happy,” “like,” and “surprise”) whereas negative-parenting-style-type students tended to express more negative emotions (i.e., “sad,” “fear,” and “hate”) in their posts. We used the Chinese Affect Lexicon (16) including 27,466 words from seven emotional categories, i.e., “happy” (1,967 words), “like” (11,108 words), “surprised” (228 words), “angry” (388 words), “sad” (2,314 words), “fear” (1,179 words), and “hate” (10,282 words). Table 2 lists the mean proportions of emotional words used by students under the three types of parenting styles. We conducted a student's t-test and verified that the differences between different types of student were significant (*p* < 0.01) in the emotional categories “happy,” “like,” and “sad.” As the significant predictor of suicidal ideation, more attention should be paid to the negative parenting style type (17, 18).

**Table 2:** The mean proportions of emotional words used by 575 student users under the parenting style types “Positive,” “Mixed,” and “Negative.”

		**Positive emotion**			**Negative emotion**			
**Parenting style type**	**Gender**	**Happy**	**Like**	**Surprise**	**Angry**	**Sad**	**Fear**	**Hate**
Positive	Male (*n* = 175)	7.53	3.52	0.06	0.24	0.23	0.05	0.72
	Female (*n* = 204)	6.11	3.93	0.09	0.17	0.38	0.09	0.90
	#All (*n* = 379)	**6.84**	3.72	0.08	**0.21**	0.30	0.07	0.82
Mixed	Male (*n* = 95)	6.24	4.39	0.09	0.13	0.28	0.11	0.89
	Female (*n* = 67)	5.33	3.92	0.06	0.24	0.50	0.14	0.92
	#All (*n* = 162)	5.95	**4.24**	**0.09**	0.16	0.35	0.12	0.90
Negative	Male (*n* = 5)	6.81	1.92	0.07	0.09	0.12	0.01	0.36
	Female (*n* = 11)	4.00	3.91	0.07	0.19	0.64	0.15	1.35
	#All (*n* = 16)	4.54	3.52	0.07	0.17	**0.54**	**0.13**	**1.16**

All values are percentages (%). In each emotional category, the maximum proportion among the three types of students is shown in bold.

From Table 2, we further observe that under the negative parenting style type, there is a significant difference (*p* < 0.01) in the emotional expression between male and female students. Female students tend to express more negative emotions (i.e., “sad,” “fear,” and “hate”) than male students. Under the negative parenting style type, the female students' most common negative emotion is hate. Moreover, we conducted a t-test to investigate the effect of gender on negative emotional expressions. As shown in Table 3, under the negative parenting style type, the effect is more significant than that under a positive or mixed parenting style type.

**Table 3:** The difference in the expression of negative emotions between male and female students under the positive, mixed, and negative parenting style types.

**Parenting style type**	**Emotion**	**Male–female**	***T*-test**
Positive	Angry	0.07	1.82 (NS)
	Sad	–0.15	–3.83^***^
	Fear	–0.04	-1.83 (NS)
	Hate	–0.18	-2.27 (NS)
Mixed	Angry	–0.13	0.95 (NS)
	Sad	–0.22	–2.05^*^
	Fear	–0.03	–2.67^**^
	Hate	–0.03	–2.48^*^
Negative	Angry	–0.10	0.60 (NS)
	Sad	–0.52	–1.20^***^
	Fear	–0.14	–1.93^***^
	Hate	–0.99	–1.20^***^

NS, non-significant.

^*^p < 0.05.

^**^p < 0.01.

^***^p < 0.001.

Overall, the differences in discourse among microblog university student users with different parenting styles indicate that it is feasible to assess parenting styles based on students' language patterns. These variations in students' expressions provide valuable insights, allowing us to focus on specific aspects when evaluating their parenting styles. In the next chapter, we incorporate these discourse differences into the construction of our assessment method to improve its accuracy and reliability.

### 2.2 Microblog-based parenting style assessment

**Figure 4:**
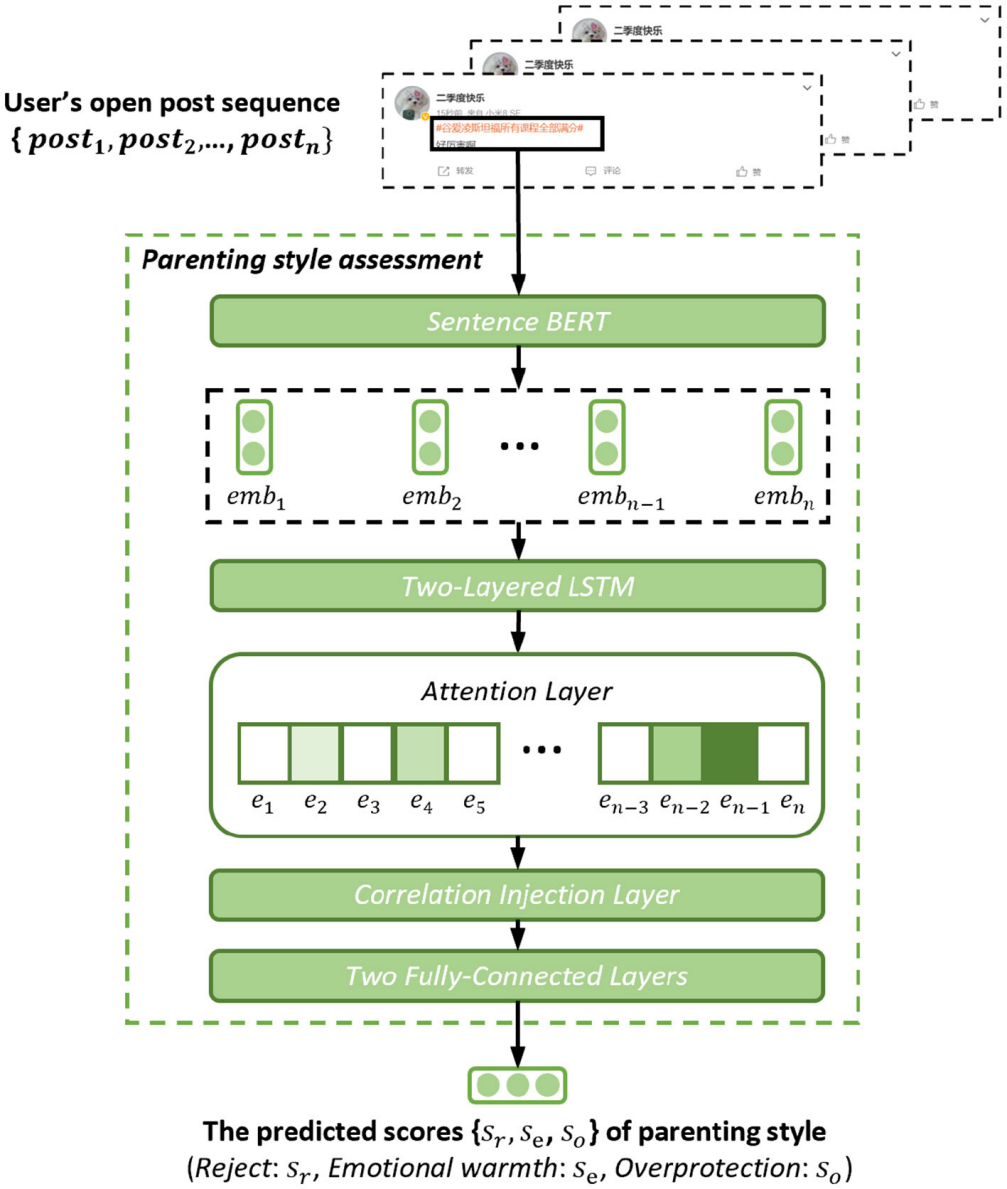
The architecture of the parenting style assessment method. In the attention layer, a deeper color indicates a higher attention weight.

In this subsection, we propose the microblog-based parenting style assessment method. Figure 4 shows the architecture of the parenting style assessment method. Given the student's open post sequence {*post*_1_, *post*_2_, ⋯ , *post*_*n*_}, the aim of the method is to predict the student's parenting style scores {*s*_*r*_, *s*_*e*_, *s*_*o*_} on the three dimensions (i.e., *Rejection, Emotion warmth, Overprotection*), where 1 ≤ *s*_*r*_, *s*_*e*_, *s*_*o*_ ≤ 4, *n* is the number of posts. Because the parenting styles of 14 (2.5%) students' parents do not fall into the category of positive, mixed, or negative, this method assesses the scores directly instead of performing three-class classification.

As illustrated in Figure 4, we first feed the student's open post sequence {*post*_1_, *post*_2_, ⋯ , *post*_*n*_} into Sentence BERT (14) to capture the linguistic and emotional information from student's discourses. The Sentence BERT obtained good performance in linguistic and emotional information extraction from texts (14, 19). The output of Sentence BERT is the embedding sequence with rich linguistic and emotional information. The sequence is represented as {*emb*_1_, *emb*_2_, ⋯ , *emb*_*n*_}, where $emb_{i}\in {\mathbb{R}}^{1\times 384}$ is the embedding vector of the *i*-th post *post*_*i*_:

(1) $$\begin{array}{l} \left\{emb_{1},\cdots \;,emb_{n}\right\}=SentenceBERT\left(post_{1},\cdots \;,post_{n}\right). \end{array}$$

Given the embedding sequence, a two-layered LSTM is employed to sense the relationship between consecutive posts. Since the student may intermittently reveal experiences related to his or her parenting style in the post sequence, LSTM can connect this valuable information together for subsequent in-depth analysis.

(2) $$\begin{array}{l} h_{i}^{1}=LSTM1\left(emb_{i},h_{i-1}^{1}\right),\\ h_{i}^{2}=LSTM2\left(h_{i}^{1},h_{i-1}^{2}\right), \end{array}$$

where $h_{i}^{1},h_{i}^{2}\in {\mathbb{R}}^{1\times 300}$ are the hidden states of LSTM1 and LSTM2 in step *i*, $H=\left\{h_{1}^{2},h_{2}^{2},\cdots \;,h_{n}^{2}\right\}\in {\mathbb{R}}^{n\times 300}$ is the output sequence.

As attention mechanism has demonstrated great capacity in key information extraction (20, 21). An attention mechanism is then applied to *H* to find the key posts related to parenting style.

(3) $$\begin{array}{l} Att=Tanh\left(H\times W_{1}+b_{1}\right)\;\in {\mathbb{R}}^{n\times 1},\\ H^{\prime }=Att^{T}\times H\;\in {\mathbb{R}}^{1\times 300}, \end{array}$$

where *Att* = {*e*_1_, *e*_2_, ⋯ , *e*_*n*_} is a sequence of attention weights paid to {*post*_1_, *post*_2_, ⋯ , *post*_*n*_}. A higher value of *e*_*i*_ means that *post*_*i*_ is more related to the parenting style of the parents of the student. *H*′ is the output of the attention mechanism, containing the key discourse information related to the parenting style of the student's parents. $W_{1}\in {\mathbb{R}}^{300\times 1}$ and $b_{1}\in {\mathbb{R}}^{1\times 1}$ are learnable parameters.

#### 2.2.1 Correlation injection module

**Figure 5:**
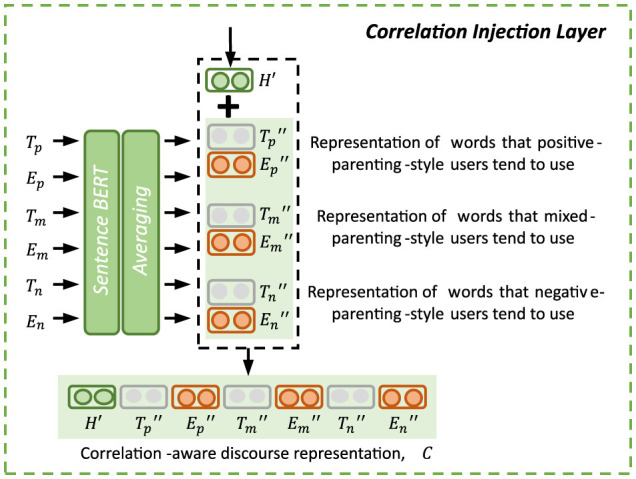
The architecture of the correlation injection layer in the parenting style assessment method.

To obtain a better performance in parenting style assessment, we designed a tailor-made module (Figure 5), injecting the found correlations into the assessment method. These correlations are that students who grew up in different parenting styles have different tendencies in linguistic and emotional expressions. This insight led our method to focus on words related to positive, mixed, and negative parenting styles, allowing our method to achieve more accurate parenting style predictions.

According to the respective tendencies of the three types of students, we created three topic word sets (*T*_*p*_, *T*_*m*_, *T*_*n*_) and three emotional word sets (*E*_*p*_, *E*_*m*_, *E*_*n*_), respectively. For instance, *T*_*n*_ contains 497 topic words that negative-parenting-style students tend to use, that is, words related to topics like “health,” “death,” and “love.” The statistical information of word sets *T*_*p*_, *T*_*m*_, *T*_*n*_, *E*_*p*_, *E*_*m*_, *E*_*n*_ is shown in Table 4. All words are from the Simplified Chinese Linguistic Inquiry Word Count (15) and the Chinese Affect Lexicon (16).

**Table 4:** The statistical information of word sets *T*_*p*_, *T*_*m*_, *T*_*n*_, *E*_*p*_, *E*_*m*_, and *E*_*n*_, which were used in the correlation injection module.

**Word sets**	**Number of words**	**Topics/emotions**	**Related parenting styles**
*T*_*p*_	4,720	Social, family, friend, work,	Positive
		Leisure, money, psychology	
*E*_*p*_	2,355	Happy, angry	positive
*T*_*m*_	301	Religion	Mixed
*E*_*m*_	11,336	Like, surprise	mixed
*T*_*n*_	497	Health, death, love	Negative
*E*_*n*_	13,775	Sad, fear, hate	Negative

Let $T_{n}^{\prime }\in {\mathbb{R}}^{497\times 300},E_{n}^{\prime }\in {\mathbb{R}}^{13775\times 300}$ be the word embedding sets of all words in *T*_*n*_ and *E*_*n*_. All the word embeddings were calculated by Sentence BERT (14). Let $T_{n}^{\prime \prime }\in {\mathbb{R}}^{1\times 300},E_{n}^{\prime \prime }\in {\mathbb{R}}^{1\times 300}$ be the average of all word embeddings in $T_{n}^{\prime }$ and $E_{n}^{\prime }$, respectively. $T_{n}^{\prime \prime }$ and $E_{n}^{\prime \prime }$ can be seen as the representations of topic and emotional words that negative-parenting-style students tend to use. We used the same approach to generate $T_{p}^{\prime \prime }\in {\mathbb{R}}^{1\times 300}$, $E_{p}^{\prime \prime }\in {\mathbb{R}}^{1\times 300}$, $T_{m}^{\prime \prime }\in {\mathbb{R}}^{1\times 300}$, and $E_{m}^{\prime \prime }\in {\mathbb{R}}^{1\times 300}$.

To inject the found correlations into the assessment method, we concatenated the discourse information *H*′ and the six representations, generated the correlation-aware discourse representation *C*:

(4) $$\begin{array}{l} C=H^{\prime }||T_{p}^{\prime \prime }||E_{p}^{\prime \prime }||T_{m}^{\prime \prime }||E_{m}^{\prime \prime }||T_{n}^{\prime \prime }||E_{n}^{\prime \prime }\in {\mathbb{R}}^{1\times 2100}, \end{array}$$

where || is the concatenate operation. *C* contains not only key discourse information (*H*′) but also the correlation information between discourse and parenting style (e.g., $T_{n}^{\prime \prime }$), which allows our method to more accurately evaluate the parenting style of the student's parents.

Finally, given the correlation-aware discourse representation *C*, the assessment method needs to deeply understand the student's discourse information and refer to the correlation information between the discourse and the parenting style to give the final parenting style assessment. Two fully-connected layers were utilized in this step:

(5) $$\begin{array}{l} \;U=Tanh\left(C\times W_{2}+b_{2}\right)\in {\mathbb{R}}^{1\times 128},\\ \left\{s_{r},s_{e},s_{o}\right\}=Tanh\left(R\times W_{3}+b_{3}\right), \end{array}$$

where *U* is the intermediate result, $W_{2}\in {\mathbb{R}}^{2100\times 128}$, $W_{3}\in {\mathbb{R}}^{128\times 3}$, $b_{2}\in {\mathbb{R}}^{1\times 128}$, and $b_{3}\in {\mathbb{R}}^{1\times 3}$ are learnable parameters. *s*_*r*_, *s*_*e*_, *s*_*o*_ are the predicted scores of the parenting style of the parents of the student in the dimensions *Rejection, Emotional Warmth*, and *Overprotection*. Higher scores indicate higher levels. In this step, we tried single-layer, two-layer, and three-layer fully-connected layers respectively, and found that the assessment performance of two-layer was the best.

## 3 Results

### 3.1 Effectiveness of the parenting style assessment

We used Mean Square Error (MSE) and Mean Absolute Error (MAE) to evaluate the performance of the parenting style assessment method. The MSE and MAE are defined as follows:

(6) $$\begin{array}{l} MAE=\frac{1}{n}\overset{N}{\underset{i=1}{\sum }}|y_{i}-y_{i}^{\prime }|,\\ MSE=\frac{1}{n}\overset{N}{\underset{i=1}{\sum }}{\left(y_{i}-y_{i}^{\prime }\right)}^{2}, \end{array}$$

where $y_{i}^{\prime }$ is the predicted score of the parenting style in the dimensions *Rejection, Emotional warmth*, and *Overprotection*. *y*_*i*_ is the label acquired from the questionnaire, and *N* is the number of students. We normalized *y*_*i*_ from 1 ≤ *y*_*i*_ ≤ 4 to −1 ≤ *y*_*i*_ ≤ 1 by the following equation:

(7) $$\begin{array}{l} y_{i}=\frac{y_{i}-2.5}{1.5} \end{array}$$

Moreover, the hyper-parameter settings of the parenting style assessment method are shown in Table 5. For each student, we selected his/her last 100 posts as input.

**Table 5:** The hyper-parameter settings of the parenting style assessment method.

**Hyper-parameter**	**Parenting style**
	**Assessment method**
Batch size	64
Learning rate	0.0001
Hidden units in LSTM	300
Number of training epochs	30

**Figure 6:**
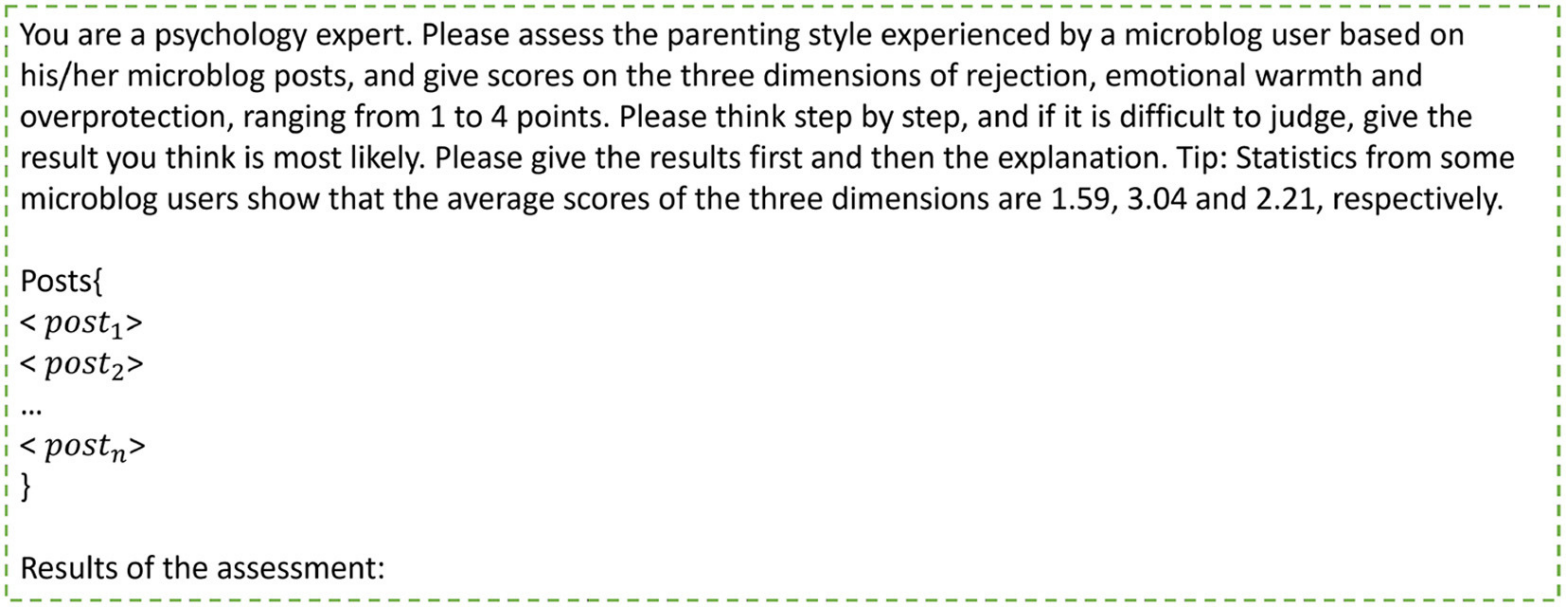
The input prompt for ChatGPT-4. < *post*_*i*_ > is the *i*-th post from the student's open post sequence.

As there is no available parenting style assessment method in the past literature, we compared our method with the following good methods in Natural Language Processing (NLP):

- TF-IDF feature + regression: this method extracts TF-IDF features from students' posts and applies a ridge regression model to assess parenting styles. In the implementation, all posts of a student were merged into a long document. Each document was extracted into a feature vector through the TF-IDF algorithm. We trained three regression models for each dimension. The training and validation sets were used to train the regression models and the testing set was used to evaluate the models.
- Handcrafted feature + ensemble model: this kind of methods are common and effective in many NLP tasks, such as depression detection (12, 22). In this study, we extracted linguistic and emotional features from students' discourses and applied an ensemble model (GBDT) to assess parenting styles. The features are the frequencies of 11 types of topic words (15) and seven types of emotional words (16) used by the students. We trained three ensemble models for the three dimensions.
- BERT embeddings + regression: this method first used the BERT model to generate the word embeddings for each word in a post, then averaged the word embeddings into a post embedding. Last, all the post embeddings were averaged into a student embedding. The student embedding was fed into a ridge regression model for parenting style assessment. Also, we trained three regression models for the three dimensions.
- BERT embeddings + LSTM + FC: this method used BERT model for post embedding generation. A sequence of post embeddings was fed into an LSTM model for deep feature extraction. Last, we added two fully-connected layers for parenting style prediction. The method predicted scores on all three dimensions simultaneously.
- BERT embeddings + CNN + FC: this method performs well in many mental health-related tasks, like depression detection (23). It first obtained the post embeddings from students' posts through the BERT model, then predicted the students' parenting style scores by a CNN model and fully-connected layers. Like the above method, this method can give scores for all three dimensions simultaneously.
- ChatGPT-4: As ChatGPT performs well in many NLP tasks (24), we selected it as a baseline method. We constructed a prompt (Figure 6) for each student in the testing set and sent it to ChatGPT-4, so that ChatGPT-4 could assess the parenting style of the student's parents.
- Questionnaire-based human judgment method: this method is a traditional assessment method. A human expert instructed students to complete the s-EMBU-C questionnaire. Our dataset also used this method to label the parenting styles of students' parents.

As illustrated in Table 6, our method outperforms all baseline methods in parenting style assessment, except the human judgment method, achieving a 0.12 MSE and 0.28 MAE. The difference in assessment performance demonstrates the effectiveness of our method. Although ChatGPT-4 excels in many NLP tasks, its performance is limited here due to lack of specific training on this task. The deep-learning-based methods (“BERT embeddings + LSTM + FC” and “BERT embeddings + CNN + FC”) slightly outperform the feature-based methods (“TF-IDF feature + regression” and “Handcrafted feature + ensemble model”). This is because the former methods can mine deeper features from the students' discourses. The human judgment method obtains the best results with zero error. However, this method is invasive, labor-intensive, and not scalable. Besides, all the methods get the best performance in the dimension *Overprotection*. This is likely because the score for the *Overprotection* dimension can be easily inferred from students' discourses.

**Table 6:** Performance analysis of the parenting style assessment on the 575 microblog student users.

	**Rejection**		**Emotional warmth**		**Overprotection**		**Average**	
	**MSE**	**MAE**	**MSE**	**MAE**	**MSE**	**MAE**	**MSE**	**MAE**
TF-IDF feature	0.19	0.34	0.19	0.35	0.08	0.24	0.16	0.31
+ regression								
Handcrafted feature	0.18	0.34	0.24	0.38	0.11	0.28	0.18	0.33
+ ensemble model							
BERT embeddings	0.18	0.33	0.21	0.36	0.11	0.28	0.17	0.32
+ regression								
BERT embeddings	0.21	0.36	0.18	0.35	0.10	0.26	0.16	0.33
+ LSTM + FC							
BERT embeddings	0.17	0.34	0.17	0.34	0.07	0.23	0.14	0.30
+ CNN + FC								
ChatGPT-4	0.35	0.45	0.33	0.48	0.30	0.41	0.33	0.45
Questionnaire-based								
Human judgment	0.	0.	0.	0.	0.	0.	0.	0.
Method								
Our method	**0.15**	**0.31**	**0.16**	**0.33**	**0.06**	**0.21**	**0.12**	**0.28**

### 3.2 Ablation study on parenting style assessment method

To comprehensively examine the impacts of the attention layer and correlation injection layer on the assessment of parenting style, an ablation test was conducted. This test removed these two layers from the method respectively, observing alterations in performance with respect to parenting style assessment. The data depicted in Table 7 highlights that the exclusions of these two layers from the method result in improvements in the Mean Square Error (MSE) of the method's performance in parenting style assessment, with errors increasing from 0.12 to 0.18 or 0.22, respectively. The increases in assessment error mean that both layers are significant and the correlation injection layer plays a more important role in the assessment process, which provides the key correlations between discourses and parenting styles.

**Table 7:** Ablation test on parenting style assessment method. “w/o” means “without.”

**Dimension**	**Our method**	**#w/o attention layer**	**#w/o correlation injection layer**
	**MSE**	**MSE**	**MSE**
Rejection	0.15	0.19 (+0.04)	0.23 (+0.08)
Emotional warmth	0.16	0.21 (+0.05)	0.23 (+0.07)
Overprotection	0.06	0.15 (+0.09)	0.20 (+0.14)
Average	0.12	0.18 (+0.06)	0.22 (+0.10)

### 3.3 Error analysis on parenting style assessment method

Within the task of parenting style assessment, we screened out 10 students from the 50 students in the test set, whose parents' parenting style scores and the assessed results by our method were significantly different. Subsequent analysis highlighted the primary factors contributing to these differences:

- Insufficient number of posts: Seven of the 10 students wrote fewer than three open posts within a year. This lack of semantic information constrains the ability of parenting style assessment method to precisely deduce the parent styles of students' parents, consequently decreasing the accuracy of assessment.
- Quotation: 4% of the 10 students incorporated quotations or paraphrased content from others' experiences and utterances, which although unrelated to the students themselves, misled our methodology into inferring incorrect parenting styles.

### 3.4 Case study

**Figure 7:**
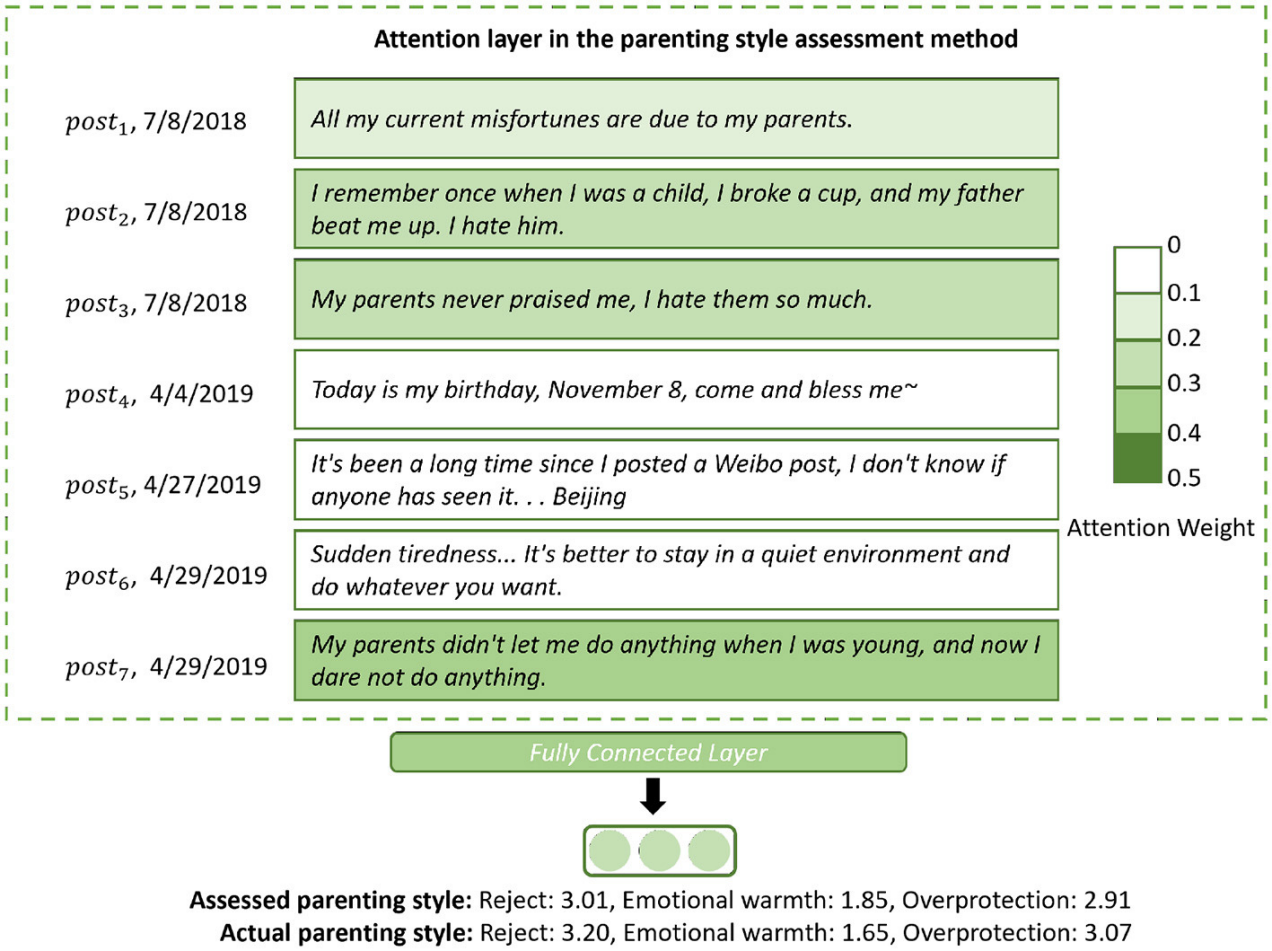
Case study: an example to show the attention weight distribution in the attention layer.

We conducted a case study (Figure 7) to show how the attention layer of the parenting style assessment method works on the student's open post sequence (discourses).

Figure 7 shows a student's open post sequence {*post*_1_, *post*_2_, ⋯ , *post*_7_}. The green dotted box represents the attention layer in the parenting style assessment method. Let $Att^{p}=\left\{e_{1},e_{2},\cdots \;,e_{7}\right\}$ be the corresponding attention weight sequences, (0 ≤ *e*_*i*_ ≤ 1, $\overset{n=7}{\underset{i=1}{\sum }}e_{i}=1$). A deeper color means a higher attention weight *e*_*i*_ paid to the *post*_*i*_.

The parenting style assessment method pays attention to *post*_1_, *post*_2_, *post*_3_, and *post*_7_, which contain descriptions of the parenting style experienced by the student (green dotted box in Figure 7). The student states that when she was young, her parent beat her for little things, never praised her, and did not let her do anything that she wanted. On the basis of the descriptions, the parenting style assessment method predicts high scores in the dimensions *Rejection* and *Overprotection* and a low score in the dimension *Emotional warmth*. The predicted scores indicate that the student experienced a negative parenting style in childhood. The slight difference between the assessed scores and the actual scores suggests that the attention layer significantly contributes to the assessment of parenting style.

### 3.5 Significance, new knowledge and research implication

#### 3.5.1 Significance

In theoretical significance, our research holds great theoretical importance in the field of parenting style assessment. By leveraging social-media-based natural language processing techniques, it challenges the traditional research paradigm that mainly relies on questionnaires and interviews. This novel approach provides a more naturalistic and large-scale data source for studying parenting styles. It enriches the theoretical framework by enabling a more in-depth exploration of the relationship between parents' implicit parenting behaviors (reflected in students' discourses on social media) and the established parenting style dimensions of *Rejection, Emotional Warmth*, and *Overprotection*.

In practical significance, the proposed parenting style assessment method has far-reaching implications. For educational institutions, especially universities, it offers a cost-effective and scalable solution to identify students who may have experienced negative parenting styles. This early identification can help in providing timely psychological interventions, reducing the likelihood of students developing mental health issues such as anxiety and depression. In the context of family education, parents can use this method to gain insights into their own parenting styles, facilitating self-reflection and potential adjustments to create a more nurturing environment for their children.

#### 3.5.2 New knowledge

In terms of methodological innovation, we introduce a new way of assessing parenting styles. Prior to this study, most research on parenting style assessment was based on self-reported questionnaires or face-to-face interviews, which are often subject to respondent bias and limited sample sizes. Our method, on the other hand, mines students' social media posts, providing a more objective and real-time view of their perceived parenting experiences. This not only overcomes the limitations of traditional methods but also uncovers new patterns in how parenting styles are manifested in natural language.

In addition, we summarize some insights on model design. Through the ablation study and comparison with baseline methods, we have discovered the importance of certain layers in our model, such as the attention layer and correlation injection layer. The attention layer can effectively focus on the relevant parts of students' discourses related to parenting styles, while the correlation injection layer helps in establishing key correlations between the text data and parenting style dimensions. These findings contribute new knowledge on how to better extract features from text for the task of parenting style assessment.

#### 3.5.3 Research implications

For future research, our error analysis indicates several directions for future research. Given that the number of posts and the presence of quotations affect the assessment accuracy, future studies could focus on developing more advanced sampling techniques to deal with limited data and more sophisticated text-filtering algorithms to exclude non-relevant information. Additionally, exploring how to integrate other types of data sources (such as multimedia content on social media) with text data may further improve the assessment performance.

For practical applications, schools and universities can incorporate this assessment method into their routine mental health screening programs. Teachers and counselors can use the assessment results to design personalized support plans for students who have experienced negative parenting styles. In the family context, parents can be educated about the importance of positive parenting styles based on the assessment results, and provided with resources and guidance on how to improve their parenting skills.

## 4 Discussion

In this study, we explored the correlations between university students' discourses on microblog and parents' parenting styles. Previous studies have demonstrated the correlations between people's behavior and their parents' parenting styles (25–27). Specifically, negative parenting styles, characterized by a lack of care and support or by overly strict control, are often associated with lower self-esteem, emotional difficulties, and antisocial behaviors in children (26, 27). However, there is no research that examines the connection between university students' discourses on microblog and their parenting styles.

In contrast to existing studies, our research extends this area by focusing on the discourse of microblog student users. Analyzing the open posts written by the 575 students, we observe that the three parenting styles affect linguistic expression and emotional expression. Negative-parenting-style students tend to use more negative emotional expressions. This may be because the negative parenting style is strongly associated with anxiety, depression, and suicide (7, 8), and individuals feeling anxiety, depression, and suicide tendencies tend to express more negative emotions on social media (28–31). The effect of sex on negative emotional expression was also investigated. According to the psychological theory (32), women express more emotions about fear and sadness than do men. We obtained similar findings that under the negative parenting style, female students express more negative emotions, such as sadness, fear, and hate. Besides, the effect is greater than that under the positive or mixed parenting style. The differences in discourse among microblog students who grew up with different parenting styles indicate that it is feasible to assess parenting styles based on discourse.

In this study, we propose the first parenting style assessment method based on the student's discourse on microblog. Previous works have shown that the rich linguistic expressions and emotional expressions posted by microblog users can help to build deep learning methods to assess their behavior and mental states, such as cyberbullying (33), depression (12), and stress (34). Inspired by this, we built a microblog-based parenting style assessment method. Experimental results on the 575 students show that the designed parenting style assessment method is effective. The parenting style is one factor of suicide (7, 8), the designed parenting style assessment method further helps to build assessment methods for other suicide risk factors, such as perfectionism (35), neuroticism (36), and ruminant thought (37). The early assessment of suicide risk factors can help prevent suicide and contribute to human wellbeing.

### 4.1 Implications

#### 4.1.1 Theoretical implications

Our research advances the understanding of how parenting styles are reflected in the linguistic and emotional expressions of students on microblogs. The novel contributions of our work are shown in following:

- Exploration of parenting styles and student discourse: few of the previous references have examined the correlations between students' discourses on microblog and parents' parenting styles. In this study, we found that positive, mixed, and negative parenting styles affect linguistic expression and emotional expression in microblog discourse. Under the negative parenting style, the effect of sex on negative expressions is greater. These findings contribute to the studies of parenting style, sex differences, and emotional analysis.
- Deep learning for parenting style assessment: we verified the effectiveness of deep learning techniques in parenting style assessment on microblogs and extended the use of microblog data in the field of students' mental health assessment. Unlike traditional parenting style assessment methods mainly based on professional questionnaires and face-to-face interviews, we proposed a microblog-based non-invasive assessment method. The new method is based on the deep learning technique and can be applied to students on a large scale at low cost. The experimental results show that the proposed parenting style assessment method effectively predicts the students' parenting styles with an MSE of 0.12 and MAE of 0.29.
- Contribution of a parenting style dataset: we constructed the first social-media-based parenting style dataset containing 111,258 open posts made by 575 students from November 1, 2019 to October 31, 2022. The collected parenting style dataset contributes to the fields of computer science and mental health assessment, offers a foundation for future studies exploring the intersection of social media use and psychological wellbeing.

#### 4.1.2 Practical implications

Our research also carries significant practical implications, particularly in the development of tool for assessing parenting styles through microblogs. This tool offers:

- Support for healthcare institutions: we proposed the first assisting tool that could be used by healthcare institutions to identify students' parenting styles. It facilitates the identification of suicide risk factors among microblog student users, and enables timely interventions to prevent suicides, which enhances human wellbeing and saves lives.
- Risk of misuse: however, this tool may be misused by organizations to conduct personalized political propaganda. For instance, an organization collected the personal data of more than 50 million Facebook users and analyzed their personalities and preferences. The analysis results were used to personalize advertisement recommendations and thus affect voter choices (38). The government should restrict the use of the proposed methods to protect the public's privacy.

### 4.2 Ethical considerations

Mental-health-related research must consider the ethical implications. The collected parenting style dataset only contains the students' discourses from their open posts. All 575 students knew of and agreed to our collection of their microblog data. The dataset was anonymized before being shared. To obtain permission to use the dataset, an applicant needs to sign an agreement that (1) the dataset cannot be used for commercial purposes; (2) the applicant cannot contact the students in the dataset; and (3) the dataset is used in accordance with local laws.

### 4.3 Limitations and future works

In this study, although promising assessment performance was achieved using the proposed parenting style assessment method, there remain issues relating to the application in real scenarios, which need further investigation.

One limitation is that during data collection, we did not gather information about how much time had passed since the student's childhood, nor did we explore whether age could serve as a moderator of negative experiences. These factors may influence the assessment, and addressing them would improve the method's accuracy.

In this study, we trained the assessment method on the fixed dataset. This method needs to gain the ability to learn new information generated by the microblog users daily in real scenarios. Over time, the assessment performance of the method will degrade. Thus, a continuous learning technique (39) should be integrated into the assessment method to retain good performance in real scenarios. In this manner, the method learns new knowledge from the incoming data and remembers essential expertise learned from the old data.

## 5 Conclusion

In this study, we explored to use microblog data to assess the parenting styles of students' parents. We (1) first investigated the correlation between students' microblog discourses and parents' parenting styles and (2) then devised a method to predict students' parenting styles from their microblog discourses. After analyzing 111,258 posts from 575 microblog student users, we found the differences in linguistic and emotional expressions across different parenting styles. Based on these findings, we designed a new parenting style assessment method that includes a correlation injection module. Experimental results on the 575 students show that our method outperforms all the baseline NLP methods, including ChatGPT-4, achieving good assessment performance by reducing MSE and MAE by 14 and 7% to 0.12 and 0.28, respectively. While our research introduces a new method for assessing parenting styles, there are still many challenges and limitations to address in its real-world application, and further exploration is needed.

## Data Availability

The raw data supporting the conclusions of this article will be made available by the authors, without undue reservation.
